# Cardio-Oncology: Managing Cardiovascular Complications of Cancer Therapies

**DOI:** 10.7759/cureus.51038

**Published:** 2023-12-24

**Authors:** Roshan Sharma, Jyoti Kashyap, Olusegun A Olanrewaju, Abdul Jabbar, FNU Someshwar, Hira Saeed, Giustino Varrassi, Hafiza Amna Qadeer, Satish Kumar, Asfand Yar Cheema, Mahima Khatri, Maha Wazir, Farhan Ullah

**Affiliations:** 1 Medicine, Sanjay Gandhi Memorial Hospital, Delhi, IND; 2 Medicine, Sri Balaji Action Medical Institute, Delhi, IND; 3 Pure and Applied Biology, Ladoke Akintola University of Technology, Ogbomoso, NGA; 4 General Medicine, Stavropol State Medical University, Stavropol, RUS; 5 Medicine, Jinnah Sindh Medical University, Karachi, PAK; 6 Medicine, Liaquat National Hospital and Medical College, Karachi, PAK; 7 Medicine, Federal Medical College, Islamabad, PAK; 8 Pain Medicine, Paolo Procacci Foundation, Rome, ITA; 9 Medicine, Foundation University Medical College, Islamabad, PAK; 10 Medicine, Shaheed Mohtarma Benazir Bhutto Medical College, Karachi, PAK; 11 Medicine, Services Hospital Lahore, Lahore, PAK; 12 Internal Medicine, Lahore Medical & Dental College, Lahore, PAK; 13 Internal Medicine/Cardiology, Dow University of Health Sciences, Karachi, PAK; 14 Medicine, Khyber Teaching Hospital, Peshawar, PAK; 15 Internal Medicine, Khyber Teaching Hospital, Peshawar, PAK

**Keywords:** cancer therapies, rare cancers, heart, cardiovascular, cardio-oncology

## Abstract

This narrative review explores the complex relationship between cancer medicines and cardiovascular health in the junction of oncology and cardiology, known as cardio-oncology. The study examines the historical development of cancer treatments and highlights the growing importance of cardiovascular problems in patient care. This text delves into the topic of cardiotoxicity, examining both conventional chemotherapeutic drugs like anthracyclines and more recent tyrosine kinase and immune checkpoint inhibitors. The complex molecular and cellular mechanisms that control cardiovascular problems are explained, including an understanding of how genetic predisposition influences an individual's sensitivity. The narrative expands into the crucial realm of risk stratification and evaluation, revealing advanced instruments for identifying cardiovascular risk in cancer patients. The importance of non-invasive imaging methods and biomarkers in early detection and continuous monitoring is emphasized.

The prioritization of preventive tactics emphasizes the need to take proactive measures incorporating therapies to protect the heart throughout cancer treatment. It also highlights the significance of making lifestyle improvements to reduce risk factors. The narrative emphasizes the changing collaborative treatment environment, advocating for merging oncologists and cardiologists in a coordinated endeavor to maximize patient outcomes. In addition to clinical factors, the review explores the critical domain of patient education and support, acknowledging its crucial role in promoting informed decision-making and improving overall patient well-being. The latter portions of the text anticipate and consider upcoming treatments and existing research efforts that offer the potential for the future of cardio-oncology. This review seeks to provide a detailed viewpoint on the intricate connection between cancer treatments and cardiovascular well-being. Its objective is to encourage a more profound comprehension of the subject and prompt careful contemplation regarding the comprehensive care of cancer patients who confront the intricate difficulties presented by their treatment plans.

## Introduction and background

Cardio-oncology is a developing discipline that addresses the growing occurrence of cardiovascular problems in individuals with cancer. It combines knowledge from both cardiology and oncology to provide a crucial answer to this issue. With the progress of medical technology, cancer patients are living longer [1]. Consequently, there is now a focus on understanding and managing the intricate relationship between cancer and cardiovascular disorders. This introduction explores the meaning and extent of cardio-oncology, highlighting the crucial importance of addressing circulatory problems in people undergoing cancer treatment [2]. Cardio-oncology aims to understand the complex connection between cancer and cardiovascular disorders, providing a holistic strategy for preventing, diagnosing, and treating heart problems that occur as a result of cancer treatment. The field involves a collaborative effort among cardiologists, oncologists, radiologists, and other healthcare experts from many disciplines, all working together to enhance patient outcomes [3]. Cardio-oncology is not simply a combination of fields but a proactive shift in healthcare that recognizes the interconnection between cancer and cardiovascular health.

The field of cardio-oncology encompasses more than just the first therapy period, acknowledging that cancer treatments can have both short-term and long-term effects on the circulatory system. Chemotherapy, radiation therapy, and targeted therapies, which are commonly used to treat cancer, can cause cardiotoxicity [4]. This means they can lead to various cardiovascular problems, including heart muscle malfunction and irregular heartbeats. The development of new cancer treatments, such as immunotherapies and kinase inhibitors, highlights the importance of a specialist approach to cardiovascular care for individuals with cancer [5]. The importance of managing cardiovascular problems in cancer patients cannot be exaggerated. Cancer survivors have a fresh array of difficulties as they navigate their lives after completing cancer treatment. Survivors frequently struggle with cardiovascular problems that can undermine their overall health and quality of life, such as heart failure, hypertension, and atherosclerosis [6]. Furthermore, recognizing and controlling cardiovascular difficulties are essential aspects of cancer therapy planning to enhance the effectiveness of cancer treatment and reduce the likelihood of adverse cardiac events.

Furthermore, the increase in cardiovascular morbidity associated with cancer imposes a significant financial strain on healthcare systems globally. The economic consequences go beyond the direct expenses linked to cardiovascular procedures, including indirect costs connected with extended hospital admissions, rehabilitation, and the potential decrease in productivity among cancer survivors [7]. Therefore, cardio-oncology is becoming a crucial priority for healthcare systems aiming to provide comprehensive and cost-efficient care to a growing and aging population affected by cancer.

The psychosocial ramifications of cardiovascular problems in cancer patients require consideration beyond the clinical domain. Patients and their families often feel overwhelmed by the emotional burden of managing both cancer and cardiovascular conditions [8]. Incorporating cardio-oncology into the broader context of cancer treatment acknowledges the comprehensive requirements of patients, including their physical, emotional, and social aspects. Cardio-oncology is crucial beyond conventional medical bounds, recognizing the complex relationship between cancer and cardiovascular well-being [9]. This field spans various aspects, including prevention, early detection, and intervention, providing a comprehensive strategy to address the growing cardiovascular problems in cancer patients. Understanding the importance of cardio-oncology is crucial, not only for improving patient outcomes but also for addressing the broader societal and economic impact of managing both cancer and cardiovascular disorders. Cardio-oncology is positioned at the vanguard of the changing healthcare environment, with the potential to influence the future of cancer treatment by incorporating cardiovascular well-being into the holistic care of cancer patients [10].

## Review

Methodology

The narrative review on cardio-oncology and addressing cardiovascular complications in cancer patients adheres to the Systematic Approach for Narrative Review (SANRA) guidelines. This ensures a meticulous and organized methodology to summarize the current literature impartially and thoroughly effectively. A comprehensive exploration of electronic databases such as PubMed, MEDLINE, Scopus, and Web of Science was carried out to commence this review. The search amalgamated pertinent keywords, such as "cardio-oncology," "cardiovascular complications in cancer patients," and associated topics. The inclusion criteria consisted of works that were published in English, underwent peer review, and were accessible up until the search cutoff date. The objective of the literature search was to locate original research articles, review papers, and meta-analyses that examine the complex association between cancer and cardiovascular health. The selection method entailed scrutinizing titles and abstracts to ascertain their pertinence to the domain of cardio-oncology and its importance. In addition, reference lists of selected papers were carefully examined to discover any other relevant studies, ensuring a thorough synthesis of all available information.

In the subsequent stage, data extraction was conducted using SANRA criteria to gather information from the chosen publications systematically. The data points encompassed the study's design, sample characteristics, interventions or exposures, results about cardiovascular problems in cancer patients, and significant findings. Two reviewers separately conducted the systematic extraction process to mitigate bias, resolving any inconsistencies by discussion and consensus. The selected studies underwent quality assessment utilizing standardized procedures suitable for their study designs. The Cochrane Collaboration's Risk of Bias tool was used for randomized controlled trials. At the same time, observational research was evaluated using techniques like the Newcastle-Ottawa Scale. The quality assessment sought to assess each study's internal validity and methodological rigor, which would inform the overall level of evidence.

The narrative synthesis adhered to the SANRA framework, prioritizing a methodical and clear-cut approach. The synthesis entailed arranging the gathered data into logical themes and patterns, emphasizing the present understanding of cardio-oncology and the significance of addressing circulatory problems in individuals with cancer. The synthesis approach also entailed thoroughly evaluating the evidence and recognizing any constraints and deficiencies in the existing literature. To strengthen the reliability of the narrative review, a systematic and replicable methodology was employed in formulating evidence-based arguments. By merging quantitative and qualitative investigations, the topic was fully explored, encompassing multiple views and research approaches. The results were placed in the larger healthcare context, highlighting the consequences for clinical practice, policy, and future research endeavors.

Finally, the review followed ethical guidelines, ensuring adequate source citation and preserving intellectual property rights. The ethical framework included the principles of transparency in reporting, disclosure of potential conflicts of interest, and ensuring the confidentiality of patient data as necessary. Overall, the approach used in this narrative review adheres to the SANRA standards, ensuring a systematic and thorough analysis of the current body of literature on cardio-oncology and the importance of managing circulatory problems in individuals with cancer. The systematic methodology employed for literature search, data extraction, quality evaluation, and narrative synthesis guarantees the dependability and clarity of the review process, hence enhancing the credibility and validity of the findings reported in this narrative review.

Historical perspective

The historical account of the development of cancer treatments and their effects on the cardiovascular system spans several decades, illustrating the complex relationship between improvements in cancer therapy and the growing understanding of cardiovascular problems [1]. This chronicle of events in oncology and cardiology highlights important milestones, revealing the progressive acknowledgment of cardiovascular complications linked to early cancer therapies. The origins of cancer treatment can be traced back to the early 20th century, characterized by the introduction of surgical treatments. Radical procedures, like the Halsted mastectomy for breast cancer, were early and innovative attempts to eliminate malignancies surgically [2]. During this period, there was a lack of comprehensive knowledge about cancer biology, and the focus of therapies was mostly on removing visible tumors rather than addressing the presence of microscopic disease. The documentation of the influence on the cardiovascular system during these initial surgical treatments was inadequate, as the main emphasis was on eliminating cancer using primitive and frequently disfiguring methods [3]. Radiation therapy was introduced as a widely accepted cancer treatment throughout the mid-20th century. Although radiation therapy provided a non-surgical alternative, its impact on nearby healthy tissues, such as the cardiovascular system, grew more evident over time. Previous radiation procedures did not possess the accuracy of modern technologies, resulting in unintentional radiation exposure to the heart and major blood arteries when treating thoracic and mediastinal cancers [4]. The identification of radiation-induced cardiovascular consequences, such as pericarditis and coronary artery disease, represented a crucial turning point in comprehending the cardiotoxicity of cancer treatments [4].

In the late 20th century, there was a significant change in thinking with the introduction of systemic chemotherapy. Alkylating agents, antimetabolites, and anthracyclines have significantly advanced cancer pharmacotherapy. Nevertheless, the harmful effects on the heart caused by anthracyclines, such as doxorubicin, became apparent, adding a new aspect to the relationship between cancer and cardiovascular well-being [5]. Vigilant monitoring and management are necessary to alleviate cardiac problems caused by anthracycline-induced cardiomyopathy, which is characterized by irreversible damage to the myocardium. With the advancement of knowledge in cancer biology, tailored medicines were developed during the late 20th and early 21st centuries [5]. Tyrosine kinase inhibitors (TKIs), monoclonal antibodies, and immune checkpoint inhibitors marked the beginning of a new era in precision medicine. Although these medicines significantly changed cancer treatment by focusing on specific molecular pathways, their first effects on the cardiovascular system were not completely understood [5]. The emergence of cardiovascular problems, such as hypertension, heart failure, and thrombosis, has highlighted the need for a sophisticated strategy for managing the cardiovascular health of cancer patients.

Identifying cardiovascular issues during the initial stages of cancer therapy has gained significant traction in the 21st century, primarily due to incorporating cardio-oncology as a distinct field of study. The area emerged in reaction to the increasing number of cancer survivors experiencing cardiovascular complications caused by the direct and indirect consequences of cancer treatments [6]. Cardio-oncology has emerged as a crucial connection between cardiology and cancer, highlighting the significance of healthcare providers working together to enhance patient care. In the age of individualized therapy, it has become crucial to identify and treat cardiovascular problems in cancer patients promptly [6]. The development of sophisticated imaging methods, such as echocardiography and cardiac MRI, has made it possible to detect cardiac dysfunction early, even before symptoms appear [7]. This enables prompt intervention and treatment. Healthcare practitioners have created risk stratification models and monitoring algorithms to help identify and manage the cardiovascular risks of different cancer therapies.

The historical analysis of the development of cancer treatments and their effects on the cardiovascular system demonstrates a complex relationship between scientific progress and medical difficulties. From the primitive surgical procedures of the early 20th century to the meticulousness of modern targeted medicines, the progression has been characterized by successes and obstacles [7]. Identifying cardiovascular difficulties during initial cancer therapies has led to the development of the field of cardio-oncology, which highlights the significance of a multidisciplinary strategy to enhance the cardiovascular well-being of cancer patients throughout their cancer treatment process [8]. This historical story provides a basis for comprehending the current endeavors to strike a balance between the effectiveness of cancer treatments and the maintenance of cardiovascular well-being as we persist in navigating the intricate realm of cancer and cardiovascular disorders [9].

Epidemiology of cardiovascular complications in cancer patients

The study of cardiovascular problems in cancer patients is a rapidly developing area of research that has gained significant interest due to the expanding number of cancer survivors and the changing landscape of cancer treatments [10]. This section explores the frequency of cardiovascular events in individuals who have survived cancer and the identification of populations at high risk [9]. It provides valuable insights into the intricate relationship between cancer and cardiovascular well-being.

Prevalence of Cardiovascular Events in Cancer Survivors

The incidence of cardiovascular problems in patients who have survived cancer has significantly increased in recent years, primarily due to the improved survival rates resulting from successful cancer treatments [10]. With the increasing efficacy of cancer treatments, there has been a change in attention toward comprehending and handling the enduring effects of cancer and its medicines to support survivors [11]. Cardiovascular events, such as heart attacks and heart failure, have become essential factors in the sickness and death of those who have survived cancer. Multiple studies have aimed to measure the frequency of cardiovascular events in individuals who have survived cancer, considering various populations and forms of cancer, including this systematic review and meta-analysis, which involved analyzing data from several studies [12]. The results indicated that the prevalence of cardiovascular events in cancer survivors varied between males and females, depending on factors such as the period of follow-up and the specific cancer treatments received. This thorough examination revealed the significant weight of cardiovascular issues in persons who had effectively finished cancer treatment. The incidence of cardiovascular incidents varies depending on the specific cancer treatment employed. Survivors of breast cancer who undergo treatment with anthracyclines and HER2-targeted treatments may encounter a heightened susceptibility to heart failure and cardiomyopathy [13]. Likewise, those who had radiation therapy for malignancies in the chest or middle of the chest are at risk of developing radiation-induced heart disease, which is marked by anomalies in the heart valves and the arteries that provide blood to the heart. Identifying these particular connections is essential for customizing surveillance and preventive measures to address the distinct cardiovascular risks linked to various cancer treatments [14].

Identification of High-Risk Populations

Identifying groups of cancer patients who are at high risk for cardiovascular problems is crucial for implementing specific preventative measures and individualized healthcare strategies [14]. Multiple factors contribute to identifying individuals who are at a heightened risk of experiencing cardiovascular issues either during or after undergoing cancer therapy. The age of an individual significantly influences their vulnerability to both cancer and cardiovascular disorders [15]. Older persons may have multiple simultaneous medical conditions, such as high blood pressure and diabetes, which collectively raise the likelihood of experiencing cardiovascular events. In addition, advanced age is linked to a reduced ability for the heart to recover after being exposed to specific cancer treatments, which increases the susceptibility to cardiovascular issues [16]. Cancer type and treatment regimens significantly impact the likelihood of cardiovascular events. As previously stated, breast cancer survivors who undergo treatment with anthracyclines and HER2-targeted medicines have specific cardiovascular risks that differ from those of patients with different forms of cancer [17]. Comprehending these connections enables healthcare practitioners to customize monitoring methods and preventive measures according to the distinct attributes of each patient's cancer and treatment history. Genetic predisposition has a role in the differences in cardiovascular vulnerability among cancer patients [18]. Specific genetic variations can impact the effectiveness of cancer treatments and the probability of experiencing cardiovascular problems. Ongoing endeavors are being made to clarify the genetic factors contributing to cardiotoxicity. These efforts show potential for creating risk-stratification models that integrate genetic data into clinical decision-making [19].

The existence of cardiovascular comorbidities requires a meticulous equilibrium between cancer treatment and cardiovascular risk mitigation, frequently necessitating intimate cooperation between cardiologists and oncologists [19]. Lifestyle factors, such as smoking, lack of physical activity, and unhealthy eating patterns, play a role in increasing the overall risk of cardiovascular problems in individuals who have survived cancer [20]. It is essential to incorporate survivorship care programs that specifically target lifestyle adjustments, including consistent physical activity and a diet promoting heart health, to reduce long-term cardiovascular risks effectively. Lifestyle treatments improve cardiovascular health and boost the general well-being and quality of life in individuals who have survived cancer [21]. The study of cardiovascular problems in cancer patients highlights the complex connection between cancer and cardiovascular well-being. The occurrence of cardiovascular events in individuals who have survived cancer is a result of the changing nature of cancer treatments. It highlights the importance of having a thorough grasp of long-term survival [22]. Identifying populations at high risk makes it possible to implement tailored therapies and personalized strategies for managing cardiovascular risk. Ongoing research efforts are focused on improving risk prediction models, investigating genetic factors contributing to heart toxicity, and optimizing ways of caring for survivors [23]. Healthcare practitioners can improve outcomes and enhance the quality of life of cancer survivors by adopting a multidisciplinary and patient-centered strategy to traverse the complex landscape of cancer and cardiovascular illnesses [22].

Cardiotoxicity of cancer therapies

Cardiotoxicity, a significant concern in the field of cancer treatments, refers to a range of adverse effects on the circulatory system caused by different types of therapy [24]. This section examines the cardiotoxic effects caused by chemotherapy, with a particular emphasis on specific drugs, including anthracyclines, TKIs, immune checkpoint inhibitors, and the influence of radiation therapy on the heart [25]. In addition, this article provides a comprehensive analysis of the many mechanisms that contribute to cardiovascular problems, such as the molecular and cellular pathways associated with cardiotoxicity and genetic susceptibility.

Chemotherapy-Induced Cardiotoxicity

Chemotherapy, a fundamental component of cancer therapy, is linked to various negative consequences, with cardiotoxicity being one of the most severe [25]. Chemotherapy-induced cardiotoxicity presents as both structural and functional abnormalities in the heart, resulting in diseases such as heart failure, myocardial infarction, and arrhythmias. The occurrence and intensity of chemotherapy-induced cardiotoxicity differ based on the specific medications used, total dosage, and individual patient characteristics [26]. Anthracyclines, including doxorubicin and daunorubicin, are highly effective and commonly utilized chemotherapy drugs. Their effectiveness in treating different types of malignancies is widely recognized. Still, they have a substantial risk of causing damage to the heart [26]. Anthracycline-induced cardiotoxicity commonly manifests as dilated cardiomyopathy, resulting in compromised systolic function [26].

Anthracycline-induced cardiotoxicity is caused by producing reactive oxygen species (ROS) and oxidative stress. Anthracyclines disrupt mitochondrial activity and generate ROS, harming cellular constituents, including lipids, proteins, and DNA. The observed impairment in mitochondrial activity and subsequent harm to cells are responsible for the programmed cell death of cardiomyocytes, known as apoptosis, which ultimately leads to impaired heart functioning [27]. TKIs are a class of drugs inhibiting the activity of tyrosine kinases. TKIs, a category of precise medicines, have transformed the field of cancer treatment by obstructing specific communication routes vital for tumor growth. Nevertheless, certain TKIs, such as sunitinib and imatinib, have been linked to cardiovascular problems [28]. The cardiotoxicity caused by TKI might present as hypertension, impaired left ventricular function, and, in severe instances, heart failure. TKI-induced cardiotoxicity is caused by inhibiting tyrosine kinases, which disrupt signaling pathways necessary for maintaining cardiovascular homeostasis [29]. VEGF inhibition, a frequent mechanism observed in TKIs, can result in compromised endothelial function, hypertension, and heightened vascular resistance, contributing to cardiovascular events.

Immune checkpoint inhibitors, a new type of immunotherapy, have demonstrated exceptional effectiveness in treating many malignancies. Nevertheless, they are linked to immune-related adverse effects, such as cardiovascular problems [30]. Myocarditis is an uncommon yet potentially lethal immune-related side effect associated with immune checkpoint inhibitors, including anti-CTLA-4 and anti-PD-1/PD-L1 drugs. The etiology of immune checkpoint inhibitor-induced myocarditis involves aberrant immunological responses and activation of T-cells [31]. Autoimmune-induced inflammation in the myocardium can result in myocardial damage, compromised contractility, and, in extreme circumstances, heart failure. Prompt identification and treatment of immune checkpoint inhibitor-induced myocarditis are essential to minimize unfavorable consequences [32]. Radiation therapy, a crucial element of cancer treatment, can unintentionally impact the heart, resulting in radiation-induced heart disease (RIHD). RIHD comprises a range of cardiovascular problems, such as pericarditis, coronary artery disease, and valvular anomalies [33]. The risk of radiation-induced heart disease (RIHD) is contingent upon various factors, including the cumulative radiation dose, how the radiation is administered in divided doses, and the proximity of the heart to the area being irradiated. The etiology of radiation-induced cardiovascular problems encompasses endothelial injury, inflammation, and fibrosis [34]. Radiation causes an imbalance in the delicate functioning of the endothelium, resulting in malfunction and higher permeability. Long-term inflammation and scarring occur, leading to changes in the blood vessels and heart structure [35]. Coronary artery involvement can lead to the hastened development of atherosclerosis and a heightened susceptibility to myocardial infarction.

Mechanisms underlying cardiovascular complications

Molecular and Cellular Pathways Involved in Cardiotoxicity

The molecular and cellular mechanisms underlying chemotherapy-induced cardiotoxicity are intricate and diverse. Anthracyclines trigger programmed cell death in heart muscle cells by activating pathways that depend on the protein p53. Moreover, the disturbance of iron metabolism and the production of free radicals lead to oxidative stress and impairment of mitochondrial function [24]. TKIs induce cardiotoxicity by disrupting crucial signaling pathways necessary for maintaining the balance and stability of the heart muscle. Inhibition of VEGF, a frequent mechanism observed in TKIs, disturbs the angiogenesis process and endothelial cells' functioning. This disruption leads to the development of hypertension and poor heart function [25]. Interfering with cellular processes by inhibiting other tyrosine kinases can harm cardiomyocytes. Immune checkpoint inhibitors regulate the immune response, resulting in immunological-related adverse effects. Myocarditis is an uncommon yet severe condition characterized by autoimmune-induced inflammation in the heart [25]. Myocardial damage and decreased contractility are caused by dysregulated T-cell activation and the release of proinflammatory cytokines.

Radiation-induced cardiovascular problems arise from the disturbance of endothelial function, leading to subsequent inflammation and fibrosis. Endothelial injury results in heightened vascular permeability and inflammation, triggering events that contribute to the development of chronic cardiovascular diseases [36]. Cardiac tissues undergoing fibrotic alterations may result in compromised contractility and structural problems.

Genetic Predisposition and Susceptibility

Genetic factors contribute to the variation in cardiovascular vulnerability among cancer patients receiving treatment. Genetic predisposition can impact how chemotherapeutic chemicals are metabolized and eliminated from the body, influence the effectiveness of targeted therapies, and alter the likelihood of developing cardiovascular problems [37]. Genetic factors primarily influence the cardiovascular vulnerabilities found among cancer patients undergoing treatment. Genetic variations in UGT1A1, HER2, ABCB1, TPMT, and CYP2D6 genes can affect how some chemotherapeutic medications and targeted therapies are metabolized and how successful they are. For example, changes in the UGT1A1 gene can impact the rate at which irinotecan is eliminated from the body. At the same time, differences in HER2 can affect how well HER2-targeted treatments such as trastuzumab work [37].

Furthermore, genes such as ABCB1, TPMT, and CYP2D6 are linked to the transportation and metabolism of drugs, which might impact both the drugs' effectiveness and the likelihood of experiencing cardiovascular problems. Identifying these genetic vulnerabilities is essential for customizing cancer therapies based on individual genetic characteristics, maximizing treatment effectiveness, and reducing the possibility of cardiovascular complications in cancer survivors [38]. Genetic differences in genes related to drug metabolism, including those that encode enzymes essential for anthracycline metabolism, can impact how individuals respond to chemotherapy [38]. Likewise, variations in genes linked to cardiovascular function and systems for repairing damage can influence the likelihood of developing cardiotoxicity. Comprehending the genetic factors that cause heart toxicity shows potential for personalized healthcare, enabling the detection of patients with greater susceptibility and creating customized treatments [39]. Genetic testing has the potential to become a crucial part of assessing risk levels, allowing healthcare professionals to enhance cancer treatment plans while reducing the likelihood of cardiovascular issues [40].

Ultimately, the harmful effects on the heart caused by cancer treatments offer a complex problem that requires a thorough comprehension of the underlying systems responsible for cardiovascular issues [41]. The complex relationship between cancer treatment and cardiovascular health is highlighted by chemotherapy-induced cardiotoxicity, which encompasses the effects of anthracyclines, TKIs, and immune checkpoint inhibitors, as well as the repercussions of radiation therapy on the heart [42]. The molecular and cellular mechanisms of cardiotoxicity provide insight into the intricate sequence of events that contribute to cardiovascular problems. At the same time, genetic predisposition and susceptibility emphasize the unique variations in how patients respond [43]. As we explore the changing field of cancer treatments, continuous research efforts aim to improve our knowledge of cardiotoxicity, leading to individualized strategies that enhance the effectiveness of cancer treatment while protecting cardiovascular health [44].

Risk stratification and assessment

The process of evaluating and categorizing the likelihood of cardiovascular complications in cancer patients is an essential part of providing comprehensive treatment [12]. This process aims to improve outcomes by identifying individuals at greater risk and implementing efforts to prevent and treat these issues. This section examines methods for evaluating the likelihood of developing cardiovascular disease, methods for tracking and measuring cardiovascular health, indicators in the body that can be used to measure risk, strategies for preventing heart disease, interventions that protect the heart during cancer treatment, changes to one's lifestyle that can reduce risk factors, managing factors that increase the risk of heart disease, and cooperative efforts between doctors who specialize in cancer therapy and those who specialize in heart health [13].

Tools for Assessing Cardiovascular Risk in Cancer Patients

Evaluating the risk of cardiovascular issues in cancer patients necessitates a systematic method, employing instruments that take into account the intricate interaction between cancer treatments and pre-existing cardiovascular ailments [11]. Conventional methods for evaluating cardiovascular risk, including the Framingham Risk Score, might not accurately quantify the risk in individuals with cancer because of the distinct harmful effects on the heart caused by specific cancer therapies [15]. The Cardio-Oncology Risk Score (CORS) is a novel tool developed for cancer patients that combines cancer-related characteristics, treatment methods, and conventional cardiovascular risk factors. CORS takes into account variables such as age, gender, cancer type, chemotherapy, and radiation therapy to offer a more sophisticated assessment of cardiovascular risk in this specific group [26]. These tools assist healthcare providers in identifying individuals who may require increased monitoring and preventive actions.

Monitoring Techniques and Biomarkers

Monitoring measures are essential for promptly identifying cardiovascular problems that may arise during cancer treatment. Echocardiography and cardiac MRI are non-invasive imaging techniques that can be used to evaluate the structure and function of the heart [23]. Regular heart monitoring, particularly for patients undergoing treatments that may harm the heart, such as anthracyclines or trastuzumab, helps detect any signs of heart problems before they become clinically apparent. Biomarkers are also crucial in evaluating cardiovascular health while undergoing cancer treatment [34]. Cardiac troponins and natriuretic peptides are often employed biomarkers that can signify damage to the heart muscle and strain on the cardiac system. Heightened concentrations of these biomarkers may necessitate further cardiac assessment and intervention [35]. Ongoing research is focused on incorporating new biomarkers, such as cardiac imaging-derived biomarkers or circulating microRNAs, to improve the accuracy and precision of cardiovascular monitoring in cancer patients [40].

Preventive strategies

Preventive methods are designed to minimize the risk of cardiovascular problems and decrease the occurrence of complications during and after cancer therapy [23]. These strategies involve a comprehensive plan that includes changing one's lifestyle, managing risk factors, and implementing treatments to protect the heart.

Cardioprotective Interventions During Cancer Treatment

Cardioprotective strategies seek to alleviate the detrimental impact of cancer treatments on the circulatory system. Dexrazoxane is a cardioprotective drug that is administered alongside anthracycline chemotherapy to decrease the likelihood of developing cardiomyopathy [34]. The possibility for cardioprotective therapies to maintain the anticancer efficacy of anthracyclines while preserving cardiac function emphasizes the importance of balancing cancer therapy effectiveness with cardiovascular safety [24]. Moreover, using angiotensin-converting enzyme (ACE) inhibitors and beta-blockers has demonstrated potential in averting chemotherapy-induced cardiotoxicity [25]. These drugs, frequently employed in the management of cardiovascular illness, show cardioprotective properties and could be advantageous for certain cancer patients who are at risk of developing cardiovascular problems [26].

Lifestyle Modifications and Risk Factor Management

Implementing lifestyle adjustments is crucial for reducing cardiovascular risk in cancer patients. Promoting a heart-healthy lifestyle, which encompasses consistent physical activity, a well-balanced diet, and smoking cessation, enhances overall cardiovascular well-being in patients [27]. Lifestyle therapies in cancer survivors target cardiovascular risk and improve quality of life and overall health. Risk factor management entails the management of modifiable cardiovascular risk factors, including hypertension, diabetes, and dyslipidemia [28]. Proactively controlling these risk factors frequently and utilizing pharmacological interventions as needed aid in reducing cardiovascular risk and can potentially improve the safety of cancer treatments [29].

Management of cardiovascular complications

Managing cardiovascular difficulties in cancer patients necessitates a customized strategy that takes into account the precise characteristics of the cardiovascular problem and the current cancer therapy [24]. Heart failure, for instance, may require the implementation of guideline-directed medical treatment, such as diuretics, beta-blockers, and ACE inhibitors. Immunosuppressive therapy may be necessary for myocarditis associated with immune checkpoint inhibitors [25]. To address radiation-induced cardiovascular problems, the approach may comprise the utilization of angiotensin receptor blockers (ARBs) to alleviate fibrotic alterations and the progression of coronary artery disease [30]. Close collaboration between oncologists and cardiologists is crucial for developing personalized therapy strategies that effectively target cancer and cardiovascular well-being [31].

Treatment Approaches for Cancer-Related Cardiovascular Issues

The treatment strategies for cardiovascular complications connected to cancer differ based on the particular difficulty. For instances of radiation-induced coronary artery disease, the medical options to be explored are percutaneous coronary intervention (PCI) or coronary artery bypass grafting (CABG) [5]. Treatment of anthracycline-induced cardiomyopathy may need the implementation of heart failure management measures, such as the use of pharmaceutical interventions. In more severe instances, the possibility of heart transplantation should be taken into account [7]. The utilization of sophisticated heart failure treatments, such as left ventricular assist devices (LVADs), signifies a state-of-the-art strategy in the treatment of severe chemotherapy-induced cardiomyopathy [8]. The field of cardio-oncology is actively involved in creating and improving therapy algorithms for circulatory problems connected to cancer. This approach focuses on personalization and involves multiple disciplines [8].

Collaborative Efforts Between Oncologists and Cardiologists

The collaboration between oncologists and cardiologists is crucial in guaranteeing the best possible care for cancer patients who are at risk of or currently experiencing cardiovascular problems. Frequent communication and collaborative decision-making enhance a thorough comprehension of the patient's health condition, treatment strategy, and any cardiovascular hazards [10]. Tumor boards that involve oncologists and cardiologists from different disciplines promote cooperative deliberations over treatment approaches, considering both cancer-related and cardiovascular factors. Shared decision-making frameworks facilitate the incorporation of cardiovascular risk assessments into the comprehensive treatment plan, promoting a patient-centered approach [11]. Moreover, continuous education and training for healthcare personnel in both fields enhance their comprehension of the overlap between cancer and cardiovascular well-being. Collaborative efforts will continue to be crucial in advancing research, improving guidelines, and optimizing the management of cancer patients who experience cardiovascular problems as the field of cardio-oncology progresses [12].

Ultimately, the effective management of cardiovascular problems in cancer patients relies on risk classification and assessment, monitoring techniques, preventive interventions, and coordinated efforts between oncologists and cardiologists [12]. Healthcare practitioners can detect and manage cardiovascular risks early during cancer treatment by utilizing thorough risk assessment tools, monitoring methods, and preventative measures. This method improves patient safety and leads to better cancer results and long-term cardiovascular health [13]. The dynamic field of cardio-oncology highlights the significance of continuous research and collaboration, ultimately influencing the future of tailored and comprehensive healthcare for patients dealing with the intricate overlap of cancer and cardiovascular conditions [14].

Role of imaging in cardio-oncology

Imaging plays a crucial role in cardio-oncology by facilitating the timely identification, surveillance, and treatment of cardiovascular problems in cancer patients [15]. This section examines non-invasive imaging techniques used to identify cardiac dysfunction, early identification, monitoring strategies, and the importance of multidisciplinary care in cardio-oncology [19]. Furthermore, it explores the significance of cooperation between oncologists and cardiologists, the creation of cardio-oncology clinics and teams, patient education and assistance, developing treatments, ongoing clinical trials, and research prospects [20].

Non-invasive Imaging Modalities for Detecting Cardiac Dysfunction

Non-invasive imaging is crucial for assessing heart function and anatomy in cancer patients receiving treatment. Echocardiography, a commonly employed technique, offers immediate visual representations of the heart, facilitating the evaluation of ventricular performance, valve irregularities, and fluid around the heart [22]. Furthermore, strain imaging, a more sophisticated echocardiographic approach, enables the timely identification of slight alterations in myocardial contractility, offering a vital understanding of subclinical cardiac disease. Cardiac MRI provides exceptional tissue characterization and imaging of cardiac structures [23]. It is especially beneficial in evaluating the vitality of the heart muscle, detecting regions of fibrosis or inflammation, and measuring the sizes of the ventricles and the ejection fraction. Cardiac MRI is highly beneficial for oncologists and cardiologists as it provides comprehensive insights into the effects of cancer treatments on the heart [24]. Nuclear imaging techniques, such as myocardial perfusion imaging and radionuclide ventriculography, offer insights into the flow of blood in the heart muscle and the functioning of the heart. These methods are especially significant in the context of radiation therapy, as they aid in evaluating the alterations in myocardial perfusion caused by radiation [25].

Early Detection and Monitoring Strategies

Prompt identification and surveillance of cardiac impairment are crucial elements of cardio-oncology healthcare. Pre-treatment baseline examinations serve as a benchmark for monitoring during cancer therapy [26]. Performing sequential imaging, such as echocardiograms or cardiac MRI, at frequent intervals throughout and after treatment enables healthcare practitioners to identify minor alterations in heart function immediately [27]. Biomarkers, such as cardiac troponins and natriuretic peptides, are valuable instruments for tracking the condition of the heart. Heightened concentrations of these biomarkers may suggest myocardial damage or strain on the heart, necessitating additional assessment and intervention [28]. Combining imaging and biomarker assessments improves the accuracy and precision of early detection methods in cardio-oncology.

Multidisciplinary care in cardio-oncology

A multifaceted approach to management is necessary due to the intricate nature of cardiovascular problems in cancer patients. Effective collaboration between oncologists and cardiologists is essential for optimizing treatment strategies and avoiding cardiovascular hazards [29]. Creating cardio-oncology clinics and teams improves communication and streamlines collaborative decision-making. It guarantees thorough attention to both cancer and cardiovascular concerns. The success of cardio-oncology programs relies heavily on the essential collaboration between oncologists and cardiologists [30]. Frequent contact between the two disciplines facilitates a comprehensive comprehension of the patient's overall health condition, treatment strategy, and prospective cardiovascular hazards. Tumor boards that involve oncologists and cardiologists from different disciplines offer a platform for cooperative deliberations on treatment approaches, considering both cancer-related and cardiovascular factors [31]. Oncologists and cardiologists can utilize shared decision-making frameworks to incorporate cardiovascular risk evaluations into the comprehensive treatment plan, promoting a patient-centric approach [32]. Enhancing communication channels and implementing interdisciplinary educational programs reinforce teamwork, ensuring healthcare providers are well-prepared to negotiate the intricate overlap between cancer and cardiovascular disorders [33].

The creation of specialized cardio-oncology clinics and teams is a purposeful effort to improve the level of care for cancer patients who are at risk of or are facing cardiovascular problems [35]. These specialty clinics convene oncologists, cardiologists, radiologists, and other healthcare experts with particular cardio-oncology knowledge. The cooperative nature of these teams enables smooth coordination of care and the creation of personalized treatment regimens [44]. Cardio-oncology clinics provide a centralized facility for thorough evaluations, imaging examinations, and continuous surveillance. Using a team-based strategy enables prompt interventions, provision of supportive care, and development of survivorship plans. Furthermore, these clinics function as instructional hubs for healthcare providers, promoting a culture of ongoing learning and research in the developing field of cardio-oncology [23].

Patient education and support

Disseminating knowledge to patients regarding cardiovascular risks and offering continuous assistance are essential elements of cardio-oncology care. Educating patients about the potential cardiovascular problems linked to cancer treatments and the significance of consistent cardiovascular monitoring is crucial [43]. Efforts to educate patients should highlight the cooperative endeavors between oncologists and cardiologists, underscoring the active management of their health aspects. Cardio-oncology supportive care and survival programs cater to the specific requirements of cancer survivors who are confronted with cardiovascular difficulties [30]. These programs include lifestyle counseling, psychological support, and assistance on how to manage cardiovascular risk factors. Incorporating patient education and support into cardio-oncology clinics fosters a comprehensive approach to patient care, prioritizing the treatment of cardiovascular problems and the general welfare of cancer survivors [31]. The dynamic discipline of cardio-oncology offers abundant prospects for forthcoming investigations and progress in healthcare. Novel treatments for cardio-oncology aim to create interventions that reduce the harmful effects on the heart while maintaining the effectiveness of cancer therapy [32]. Ongoing research is constantly investigating new imaging techniques and biomarkers to improve the accuracy of early detection and monitoring methods.

Cardio-oncology research is now investigating novel therapeutics to reduce the harmful effects of cancer treatments on the heart [33]. Dexrazoxane, a cardioprotective drug administered alongside anthracycline chemotherapy, exemplifies a therapeutic approach that has demonstrated potential in safeguarding cardiac function [34]. Continuing discoveries into the mechanisms of heart toxicity are facilitating the advancement of focused methods that reduce cardiovascular risks while maintaining the effectiveness of cancer treatments [35]. Continuing clinical trials and research endeavors in cardio-oncology enhance our understanding and improve treatment approaches. Investigative methods encompass innovative drugs, cutting-edge imaging methods, and biomarkers to improve risk categorization and prompt identification of conditions [35]. The collaboration of oncologists, cardiologists, and researchers from diverse disciplines is crucial for the progress of the area and the use of research findings to enhance patient treatment [44].

Ultimately, imaging plays a crucial role in cardio-oncology by facilitating the timely identification, surveillance, and treatment of cardiovascular problems in individuals with cancer. In conjunction with initiatives for early diagnosis and monitoring, non-invasive imaging techniques play a crucial role in providing comprehensive patient care [36]. Optimal treatment regimens and appropriate management of cancer and cardiovascular elements require collaboration between oncologists and cardiologists, resulting in multidisciplinary care [38]. Implementing cardio-oncology clinics, patient education, and support programs additionally augment the standard of care. Promising advancements in research and future medicines are expected to enhance the field and results for cancer patients susceptible to cardiovascular problems [39]. Continuing clinical trials and research endeavors will further influence the developing field of cardio-oncology, ultimately resulting in more tailored and efficient care methods [44].

## Conclusions

In conclusion, combining non-invasive imaging techniques, early detection methods, and multidisciplinary care in cardio-oncology provides a thorough strategy to enhance the cardiovascular health of individuals with cancer. Echocardiography, cardiac MRI, and nuclear imaging aid in promptly detecting problems, while the cooperative endeavors between oncologists and cardiologists, facilitated by specialized cardio-oncology clinics, emphasize the significance of comprehensive patient treatment. The main conclusions highlight the importance of actively assessing and categorizing risks, consistently monitoring the situation, and implementing tailored interventions, which may involve new therapies and supportive programs. To promote the field of cardio-oncology, it is crucial to raise awareness, foster collaboration between different disciplines, and integrate the latest research findings into clinical practice. Enhancing the quality of care and outcomes for cancer patients with cardiovascular problems can be achieved by implementing improved communication, standardized protocols, and patient-centered methods within the cardio-oncology framework.
